# The Importance of Coral Larval Recruitment for the Recovery of Reefs Impacted by Cyclone Yasi in the Central Great Barrier Reef

**DOI:** 10.1371/journal.pone.0065363

**Published:** 2013-06-05

**Authors:** Vimoksalehi Lukoschek, Peter Cross, Gergely Torda, Rachel Zimmerman, Bette L. Willis

**Affiliations:** 1 Australian Research Council Centre of Excellence for Coral Reef Studies, James Cook University, Townsville, Queensland, Australia; 2 School of Marine and Tropical Biology, James Cook University, Townsville, Queensland, Australia; 3 Australian Institute of Marine Science, Townsville, Queensland, Australia; 4AIMS@JCU, Townsville, Queensland, Australia; The Australian National University, Australia

## Abstract

Cyclone Yasi, one of the most severe tropical storms on record, crossed the central Great Barrier Reef (GBR) in February 2011, bringing wind speeds of up to 285 km hr^−1^ and wave heights of at least 10 m, and causing massive destruction to exposed reefs in the Palm Island Group. Following the cyclone, mean (± S.E.) hard coral cover ranged from just 2.1 (0.2) % to 5.3 (0.4) % on exposed reefs and no reproductively mature colonies of any species of *Acropora* remained. Although no fragments of *Acropora* were found at impacted exposed sites following the cyclone, small juvenile colonies of *Acropora* (<10 cm diameter) were present, suggesting that their small size and compact morphologies enabled them to survive the cyclone. By contrast, sheltered reefs appeared to be unaffected by the cyclone. Mean (± S.E.) hard coral cover ranged from 18.2 (2.4) % to 30.0 (1.0) % and a large proportion of colonies of *Acropora* were reproductively mature. Macroalgae accounted for 8 to 16% of benthic cover at exposed sites impacted by cyclone Yasi but were absent at sheltered sites. Mean (± S.E.) recruitment of acroporids to settlement tiles declined from 25.3 (4.8) recruits tile^−1^ in the pre-cyclone spawning event (2010) to 15.4 (2.2) recruits tile^−1^ in the first post-cyclone spawning event (2011). Yet, post-cyclone recruitment did not differ between exposed (15.2±2.1 S.E.) and sheltered sites (15.6±2.2 S.E.), despite the loss of reproductive colonies at the exposed sites, indicating larval input from external sources. Spatial variation in impacts, the survival of small colonies, and larval replenishment to impacted reefs suggest that populations of *Acropora* have the potential to recover from this severe disturbance, provided that the Palm Islands are not impacted by acute disturbances or suffer additional chronic stressors in the near future.

## Introduction

Disturbance is part of the evolutionary history of coral reefs, however the increasing frequency and intensity of anthropogenic and climate-related disturbances, particularly coral bleaching and altered storm regimes [1], [2], are predicted to significantly reduce coral population sizes in the next few decades [3]. Coral reefs can recover from disturbances by re-growth from remnant live coral tissue on surviving colonies [4], [5] and/or by propagation from broken fragments following storms or other high-energy events [6], [7]. However, in cases where major disturbances reduce live coral cover catastrophically and few surviving colonies or live fragments remain, larval replenishment from less severely impacted sites becomes critical for reef recovery [8], [9]. Larval replenishment is influenced by the distances over which larvae are able to disperse, with populations that routinely receive larval subsidies from external sources more likely to recover and avoid degradation [9]. In contrast, reefs that are primarily self-seeding are expected to experience significantly reduced recruitment [8] following the depletion or local extinction of reproductively mature adult colonies. If recruitment is interrupted by repeated disturbances, coral populations may fail to recover, with the consequence that reefs will undergo phase-shifts to less desirable, algal-dominated states [10], [11].

The potential for larval replenishment of coral populations following disturbance may vary among species that differ in mode of reproduction. Coral reproductive strategies fall into two broad categories, brooding corals that have internal fertilization and release mature planulae ready to settle close to parents, and broadcast-spawning corals that release gametes, typically in a timed single or bi-annual spawning event, for external fertilization at the ocean surface potentially leading to dispersal between reefs [12]–[15]. Broadcast spawning corals predominate on the Great Barrier Reef (GBR) [12], particularly species within the genus *Acropora*, the most abundant and species-rich scleractinian genus in the Indo-Pacific [16]. *Acropora* larvae become competent to settle within a few days [17]–[19] and therefore have the potential to recruit back onto their source reef when oceanographic and meteorological conditions retain water masses in the vicinity of reefs for several days [20], [21]. However, *Acropora* larvae can remain competent to settle for many months, highlighting their potential for long distance dispersal [22], [23]. The potential for both high local retention and long-distance dispersal of *Acropora* larvae is corroborated by their population genetic structures [24], [25], the scale of stock-recruitment relationships [26], and larval dispersal models [21].

**Figure 1:**
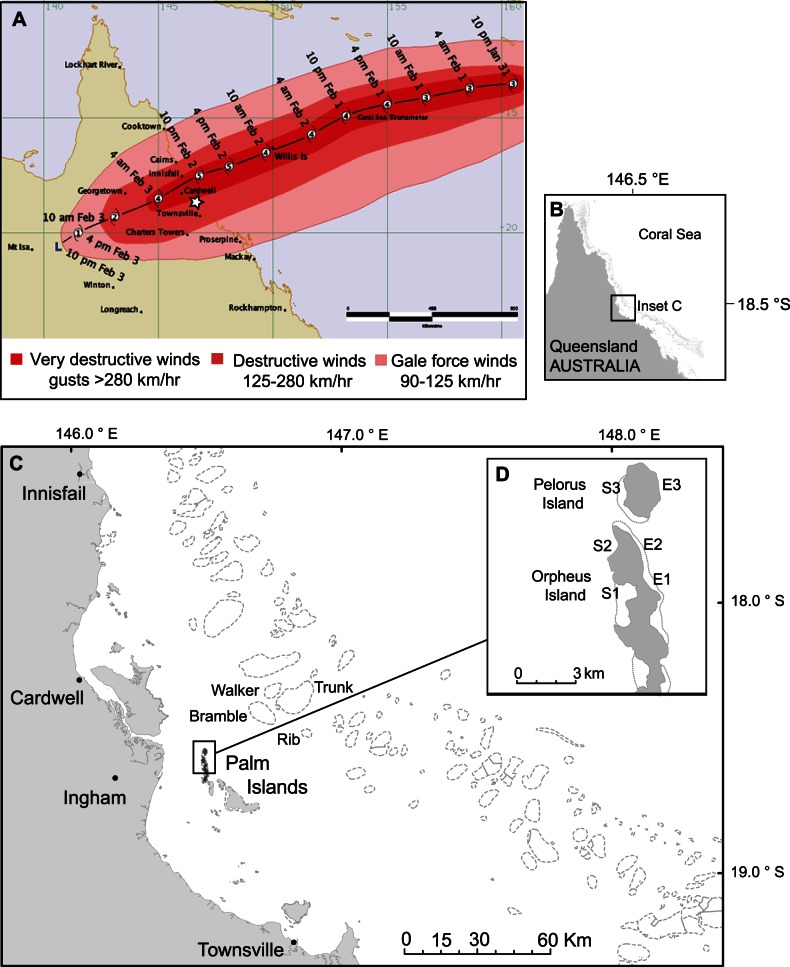
The path of severe tropical cyclone Yasi in relation to the Palm Islands, central Great Barrier Reef (GBR). *A*. Track of cyclone Yasi crossing the GBR on February 2^nd^, 2011 (source www.bom.gov.au/cyclone/history/yasi.shtml#track accessed on June 5^th^, 2012). Star indicates Palm Islands. *B*. Location of the Palm Islands on the GBR. *C.* Locations of Palm Islands and adjacent mid shelf reefs in the central GBR. *D.* Locations of exposed and sheltered study sites at Orpheus and Pelorus Islands in the Palm Islands.

The role of inter-reef larval dispersal in the replenishment of coral populations is particularly critical following disturbance events, such as severe tropical storms (known variously as cyclones, hurricanes or typhoons), which can reduce coral populations catastrophically. On February 2^nd^, 2011, severe tropical cyclone Yasi crossed the central GBR (Fig. 1a) as one of the strongest tropical storms to affect Queensland reefs since records commenced [27]; only three tropical cyclones of comparable intensity have been recorded on the GBR (one in 1899 and two in 1918) [27]. Cyclone Yasi was over 1000 km in diameter, with a central atmospheric pressure of 930 hPa, sustained wind speeds of 205 km hr^−1^, and intermittent gusts reaching 285 km hr^−1^. The Townsville wave site recorded a maximum wave height of 9.6 m (the highest recorded at the site since it commenced operation in 1975) approximately 5.5 hours before cyclone Yasi crossed the coast. The eye of cyclone Yasi passed just north of the Palm Islands, a group of inshore islands with fringing reefs in the central GBR (Fig. 1a). Rapid ecological assessments conducted in February and March 2011 indicated that many reefs in cyclone Yasi’s path experienced high levels of coral loss [28]. The branching growth forms of *Acropora* species make this group of corals among the most vulnerable to the effects of disturbances, particularly cyclones [29]. Concomitantly, this genus sustained the most significant damage by cyclone Yasi [28].

Tropical storms are among the most significant disturbances for coral reefs [30]. Indeed, recent studies demonstrated that tropical storms on the GBR accounted for 48% of coral mortality between 1985 and 2012 [31], and impacted more reefs and were responsible for larger declines in coral cover between 1995 and 2009 than coral bleaching and disease combined [32]. Impacts from storms of weak to moderate intensity are patchy over small spatial scales so recovery via the supply of larvae from non-impacted patches, combined with the re-growth of injured colonies or propagation from live fragments can be relatively rapid. However, the last three decades have seen the frequency of severe cyclones (category 3–5) almost double [33] and this trend is expected to continue as oceans warm in response to increasing levels of greenhouse gases [1], [2] (but see [27] for an alternative view). Severe tropical storms tend to kill rather than injure colonies and damage occurs over large spatial scales [30], possibly reducing the potential for and/or rate of recovery. Studies have evaluated the effects of severe tropical storms on coral diversity, abundance and community structure [29], [30], [34]–[36] and have demonstrated that severe tropical storms can reduce subsequent coral recruitment on Caribbean reefs [37]–[39]; however, there have been no studies examining the impacts that cyclones have on coral larval supply for the GBR, particularly following severe category 5 cyclones. This study quantifies the impact of cyclone Yasi on benthic communities on fringing reefs of the Palm Islands in the central GBR and compares coral larval recruitment to experimental substrata by broadcast spawning *Acropora* in the year before and after the cyclone.

## Materials and Methods

### Study Sites and Sampling Design

The study was conducted on the exposed and sheltered fringing reefs of Orpheus and Pelorus Islands in the Palm Island group, which were in the direct path of very destructive tropical cyclone Yasi (Fig. 1a). The Palm Islands are located on the inner shelf of the central section of the GBR (Fig. 1b), with the closest mid-shelf reefs (Bramble, Walker, Trunk and Rib) located approximately 20–30 km to the east and north east (Fig. 1c). A total of six study sites were sampled; three on eastern windward exposed sides (E1–E3) and three on western leeward sheltered sides (S1–S3) of Orpheus and Pelorus Islands (Fig. 1d). Sites were separated by 2 to 6 km. The four Orpheus Island sites (E1, E2, S1, S2) were in Marine National Park Zones, while the two Pelorus Island sites (E3 and S3) were in Habitat Protection Zones (http://www.gbrmpa.gov.au/__data/assets/pdf_file/0014/28112/Map6-GDA94.pdf).

Field data were collected before (2010) and after (2011) cyclone Yasi. In 2011, data for benthic cover, abundance and colony sizes for all *Acropora* species, and recruitment to experimental substrata were collected at all six sites (details below). In 2010 we were not aware that cyclone Yasi would occur so surveys were only conducted at a subset of sites before the cyclone. Table 1 provides a summary of the data collected, the survey methods used, and the sites surveyed in 2010 and 2011.

**Table 1:** Summary of field data collected before (2010) and after (2011) cyclone Yasi at Orpheus and Pelorus Islands, central Great Barrier Reef, showing the survey method used and the sites surveyed for each metric in each year (see text for details).

Metric	Year	Method	Sites
Coral cover & benthic composition	2010	N/A	
	2011	Line Intercept	E1–E3: S1–S3
Sizes of *Acropora* spp. colonies & fragments	2010	N/A	
	2011	Belt transects - size measured in field using tape measure	E1–E3: S1–S3
Colony sizes of *Acropora tenuis*	2010	Sizes estimated from corals photographed with ruler *in-situ*	E2: S1–S3
	2011	Belt transects - size measured in field using tape measure	E1–E3: S1–S3
Recruitment of *Acropora* to settlement tiles	2010	Field deployed settlement tiles	E2: S1
	2011	Field deployed settlement tiles	E1–E3: S1–S3

Permits to conduct field research in the Great Barrier Reef Marine Park were obtained from the Great Barrier Reef Marine Park Authority (GBRMPA: permit G33638.1 issued to VL). All data collected in this study can be obtained by request from VL.

### Post-cyclone Coral Cover and Benthic Composition

Benthic cover following cyclone Yasi was quantified in November 2011 using visual census of 20 m line intercept transects. At each site (E1–E3, S1–S3), six replicate transects were sampled: three on the reef crest and three on the reef slope (10 m between transects within each habitat). On each transect, the benthic category lying underneath the tape was identified, and the intercept was measured to the nearest centimeter. The following benthic categories were recorded: live hard coral, soft coral, macroalgae, rock, and other (primarily sand, rubble, sponges). Hard corals were recorded at the genus level but, because this study focuses on the spawning acroporids, coral cover is presented as Acroporidae and other hard corals. Spatial variation in benthic cover was examined using hierarchical (nested) analysis of variance (exposure, site nested within exposure). Data were log (x +1) transformed to meet the assumptions of homogenous variances.

### Post Cyclone Abundance and Sizes of Colonies and Live Fragments of Acropora spp

To quantify the impact of tropical cyclone Yasi on *Acropora* assemblages, the abundance and colony sizes of all species of *Acropora* were estimated at exposed and sheltered sites. At each site, 20 m×2 m (40 m^2^) belt transects were surveyed on the reef crest and upper slope (six replicates per site). All colonies of *Acropora* were recorded and the maximum diameter of the colony (D_1_) and the diameter at right angles (D_2_) were measured for each colony (to the nearest cm) using a tape measure. The size (mean diameter) of each colony was calculated as (D_1_+ D_2_)/2. In order to quantify the potential for recovery at impacted sites via propagation from coral fragments, the abundance and sizes of all live fragments of *Acropora* spp. were also recorded at impacted sites (E1–E3). Spatial variation in density and size of colonies was examined using hierarchical (nested) analysis of variance (exposure, site nested within exposure). Data were log (x+1) transformed to meet the assumptions of homogenous variances.

### Abundance and Colony Sizes of Acropora tenuis before and after Cyclone Yasi

Abundance and colony size data for *Acropora tenuis* at exposed and sheltered sites were collected before (2010) and after (2011) cyclone Yasi. *A. tenuis* is a broadcast spawning species that reproduces in spring on the GBR and is representative of the majority of species of *Acropora* that participate in the annual synchronised spawning event [12], [13]. Pre-cyclone sampling was conducted on SCUBA while towing a GPS that recorded the distance the diver swam every 5 seconds, to the nearest meter. The diver (VL) surveyed the crest and upper slope by systematically swimming in a zigzag pattern and thoroughly searching 2 m either side of the swim path with the aim of sampling every colony of *A. tenuis* present. Each colony of *A. tenuis* encountered was photographed with a 30 cm ruler *in-situ,* which was placed along the maximum diameter of the colony (D_1_). The ruler was used as calibration to estimate D_1_ and D_2_ to the nearest cm for colonies <30 cm and to the nearest 5 cm for colonies >30 cm. Colony size was calculated as (D_1_+ D_2_)/2. The camera and GPS clocks were synchronized and every *A. tenuis* photographed had a date and time stamp, which allowed the exact location of each colony to be mapped along the GPS path. Density was estimated by calculating the number of *A. tenuis* sampled along each 100 m of the GPS track, with the assumption that each meter swum sampled 4 m^2^ of reef area. Post-cyclone abundances and colony sizes for *A. tenuis* were estimated from the belt transect surveys used to census the abundance of the entire *Acropora* assemblage (described above). Spatial and temporal variation in the density of *A. tenuis* was examined using analysis of variance. Spatial (between exposed and sheltered sites) and temporal (pre- and post-cyclone) variation in mean colony size was tested using t-tests.

### Recruitment to Experimental Substrata

Recruitment to experimental substrata was quantified for the spawning events in the years immediately before (2010) and after (2011) cyclone Yasi. In 2010 recruitment was quantified at two sites (E2 and S1) and in 2011 recruitment was quantified at all six sites (E1–E3; S1–S3). In each year, unglazed terracotta tiles (11×11×1 cm) were attached directly to the substratum in horizontal orientation [40] on the reef crest and upper slope. Tiles were conditioned in the field for approximately one month prior to deployment. In each year, recruitment was assessed in two temporal windows: T1 spanned the first 14 days following spawning thereby sampling recruits in the ten days immediately after competency had been achieved and potentially sampling recruits spawned by local populations; T2 started at the end of T1 and spanned the subsequent month thereby sampling recruits that had spent considerable time in the water column and had likely dispersed from more distant populations. In each year, twenty conditioned tiles were deployed per site for each temporal window, i.e. 20 conditioned tiles were deployed just prior to spawning and collected two weeks post-spawning (T1), at which time 20 new conditioned tiles were deployed, which were collected at the end of T2.

Following retrieval, tiles were systematically examined (top, bottom and sides) using a dissecting photomicroscope. The availability of recruits to experimental substrata was quantified by counting all corallites (skeletal structures deposited by coral polyps), irrespective of whether or not they were alive at the time of sampling [41]; i.e. dead recruits were included in the recruitment estimates. Recruits were identified as belonging to the coral families Acroporidae, Pocilloporidae or other broadcast-spawning families following [42]. Recruits that could not be identified because they were very small or damaged were classified in a 4^th^ category labeled “unknown”. Data for brooding pocilloporids are part of a long-term recruitment study and will be reported in a separate publication. Spatial and temporal variation in recruitment was examined using hierarchical (nested) analysis of variance. Data were log (x +1) transformed to meet the assumption of homogenous variances where necessary.

### Comparison of Wind Conditions Following Spawning Events in 2010 and 2011

Wind conditions can have a major impact on surface currents and other hydrodynamic features that influence the retention and dispersal of coral larvae in the first few days after spawning [43]. To evaluate whether wind conditions differed following the 2010 and 2011 spawning events, potentially influencing spatial and temporal patterns of coral recruitment to settlement tiles, wind data were obtained from the Great Barrier Reef Ocean Observing System (GBROOS) weather station at Pioneer Bay, Orpheus Island, and from the Bureau of Meteorology (BOM) Townsville Airport Weather Station (91 km to the southwest). Cyclone Yasi destroyed the GBROOS Orpheus weather station in February 2011, so wind data for the 2011 spawning are only available from the Townsville station. Hourly wind speeds obtained for 2010 and 2011 were used to calculate daily averages for the first two weeks following spawning in each year.

## Results

### Coral Cover and Benthic Composition at Exposed and Sheltered Sites Following Cyclone Yasi

**Figure 2:**
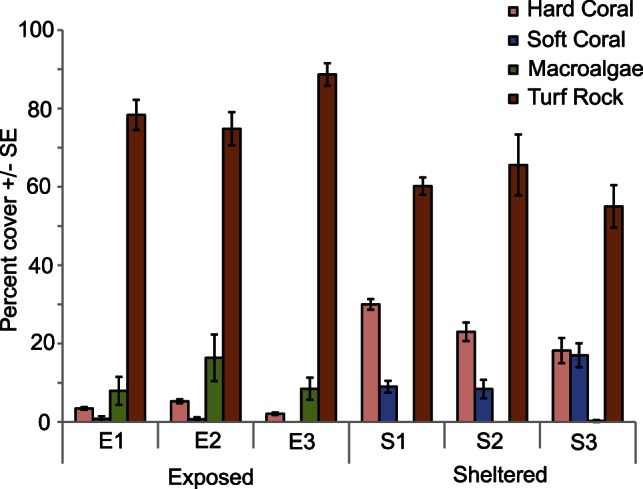
Mean (± S.E.) percent cover of hard coral, soft coral, macroalgae, and rock covered with turf alage at exposed and sheltered sites in the Palm Islands nine months after cyclone Yasi.

Mean (± S.E.) hard coral cover was significantly lower at exposed sites (F_1, 30_ = 71.6, p<0.001), where it ranged from 2.1 (0.2) to 5.3 (0.4) %, than at sheltered sites, where it ranged from 18.2 (2.4) to 30.0 (1.0) % (Fig. 2) but was not significantly different among sites within exposures (F_4, 30_ = 2.17, p>0.05). Acroporidae accounted for <0.1% of benthic cover and <1% of hard coral cover at exposed sites nine months after the cyclone. Hard corals that survived the cyclone at exposed sites were mostly encrusting or submassive non-acroporid spawning corals. Mean (± S.E.) soft coral cover was <1% at all three exposed sites, which was significantly lower than at sheltered sites (F_1, 30_ = 172.11, p<0.001), where it ranged from 8.4 (2.4) % to 17.0 (3.1) % (Fig. 2). There was no significant difference among sites within exposures (F_4, 30_ = 0.13, p>0.10). Macroalgae accounted for 8 to 16% of benthic habitats at exposed sites but were not recorded at any sheltered site (Fig. 2). Rock covered with turf algae was the dominant benthic habitat at both exposed (75–89%) and sheltered sites (55–65%) (Fig. 2).

### Post Cyclone Abundance and Sizes of Colonies and Fragments of Acropora spp

**Figure 3:**
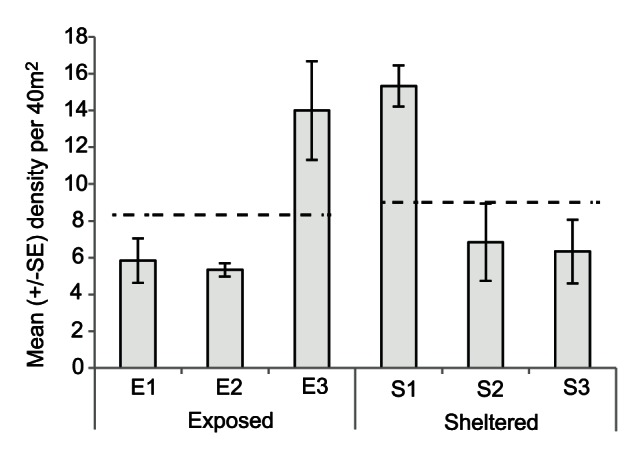
Mean density (± S.E.) of *Acropora* spp. at exposed and sheltered sites in the Palm Islands after cyclone Yasi (2011). Dashed lines indicate mean densities across the three sites in each exposure.

**Figure 4:**
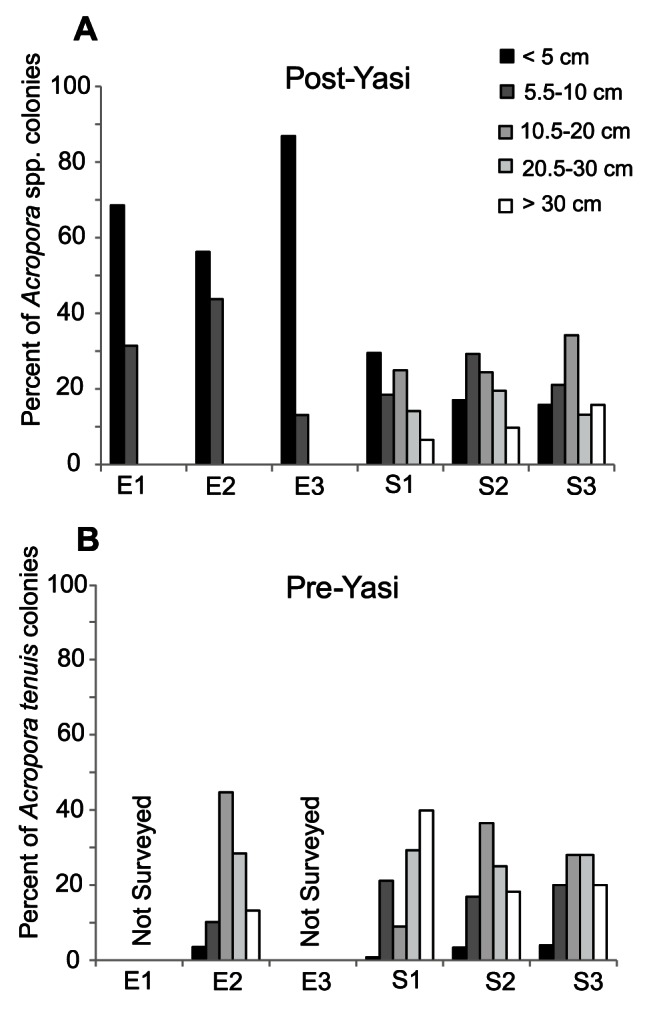
Size frequency distributions of colonies of *Acropora* in the Palm Islands. *A.* Colony size frequency distributions for all species of *Acropora* at three exposed and three sheltered sites after cyclone Yasi (2011). *B.* Colony size frequency distributions of *Acropora tenuis* at one exposed and three sheltered sites before cyclone Yasi (2010).

Mean densities of colonies of *Acropora* spp. were highest at E3 (14.0±2.7 S.E. per 40 m^2^) and S1 (15.3±1.1 S.E. per 40 m^2^), where colonies were more than twice as abundant than at any of the other four sites (Fig. 3). As such, there was a highly significant effect among sites within exposure (F_4, 30_ = 10.1, p<0.001) but no significant difference in overall density of colonies of *Acropora* spp. between exposures (F_1, 30_ = 0.8, p>0.10). However, the mean colony size of *Acropora* spp. at sheltered sites (15.2 cm ±1.2 S.E.) was almost four times larger than at exposed sites (4.3 cm ±0.2 S.E.), resulting in a highly significant difference between exposures (F_1, 30_ = 148.6, p<0.001). Specifically, all colonies of *Acropora* spp. at exposed sites were smaller than 10 cm (Fig. 4a) and typically had compact morphologies consisting of an encrusting base with a few small branches, indicating that they were small colonies (juveniles) that survived the cyclone. By contrast, at sheltered sites the size range of *Acropora* encompassed both small (juvenile) and large (adult) colonies (Fig. 4a). There were no live fragments of *Acropora* found at any of the exposed sites after cyclone Yasi.

### Comparison of Abundance and Colony Sizes of Acropora tenuis before and after Cyclone Yasi

Before the cyclone (2010), the highest abundance of *A. tenuis* was at the exposed site (E2), with a mean density of 0.69±0.05 S.E. colonies per 40 m^2^, which was significantly greater than mean densities at S1 (0.41±0.08 S.E.) and S3 (0.36±0.04 S.E.) but not at S2 (0.55±0.13 S.E.) (F_3, 20_ = 10.61, p<0.001, Tukeys HSD post-hoc tests having p<0.01). Following the cyclone (2011), there were no colonies of *A. tenuis* found at any of the three exposed sites, while mean densities (per 40 m^2^) at the sheltered sites (S1 − 0.67±0.23 S.E.; S2 − 0.50±0.37 S.E.; S3 − 0.50±0.24 S.E.) remained unchanged from pre-cyclone levels (F_1, 30_ = 0.474, p>0.10).

Before the cyclone, more than 70% of colonies of *A. tenuis* were larger than 10 cm at all sites surveyed (E2; S1–S3) (Fig. 4b) and mean colony sizes did not differ between exposures (exposed: 21.7 cm ±1.0 S.E.: sheltered: 23.6 cm ±0.8 S.E.) (t = 1.611, d.f. = 491, p = 0.108). After the cyclone, mean colony sizes of *A. tenuis* at sheltered sites (21.3 cm ±1.6 S.E.) were not significantly different from mean sizes before the cyclone (t-test = 1.407, d.f. = 372, p>0.05).

### Other Evidence of the Differential Effects of Cyclone Yasi at Exposed and Sheltered Sites

**Figure 5:**
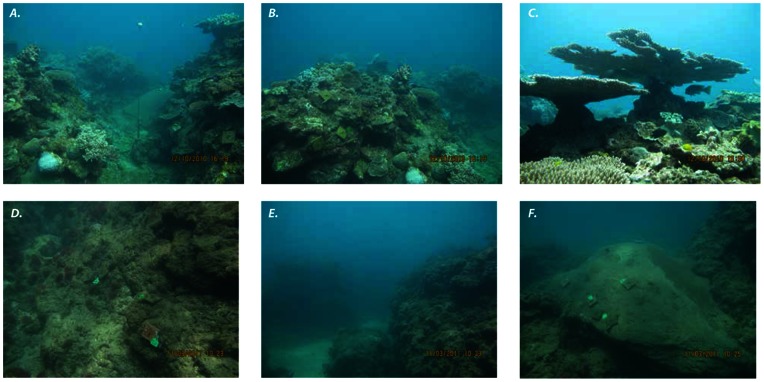
Photos of exposed study sites before (*A–C*) and after (*D–F*) cyclone Yasi showing massively reduced coral cover, benthic diversity, and structural complexity after the cyclone. Note that the settlement tiles in photos *D–F* were newly deployed following the cyclone because all of the tiles deployed before cyclone Yasi were removed by the cyclone.

Although fieldwork conducted at exposed and sheltered sites prior to cyclone Yasi did not quantify benthic cover or habitat complexity, photos taken at exposed sites before (Fig. 5 A–C) and after (Fig. 5 D–F) clearly show the impacts of the cyclone on benthic cover, coral diversity, and structural complexity. In addition, the differential impacts of cyclone Yasi at exposed and sheltered sites are demonstrated by the fact that at sheltered sites, all settlement tiles attached to the substratum before the cyclone remained *in-situ* following cyclone Yasi, whereas at exposed sites every tile deployed had been ripped out of the substratum by the cyclone and none were found.

### Recruitment to Experimental Substrata

A total of 2874 identifiable coral recruits were sampled on the settlement tiles. In 2010, 958 recruits were sampled at two sites (E2 and S1): 606 recruits in T1 (37 tiles) and 352 recruits in T2 (39 tiles). In 2011, 1916 recruits were sampled across all six sites: 940 recruits in T1 (118 tiles) and 976 recruits in T2 (117 tiles). Acroporids accounted for 94% of identifiable recruits, followed by other spawners (4%) and pocilloporids (2%). In 2010, the maximum number of recruits on a tile was 45 (at E2 in T1) and six tiles had zero recruits, while in 2011, maximum number of recruits on a tile was 29 (at E3 in T1) and four tiles had zero recruits. The following results refer to Acroporidae recruits only.

**Figure 6:**
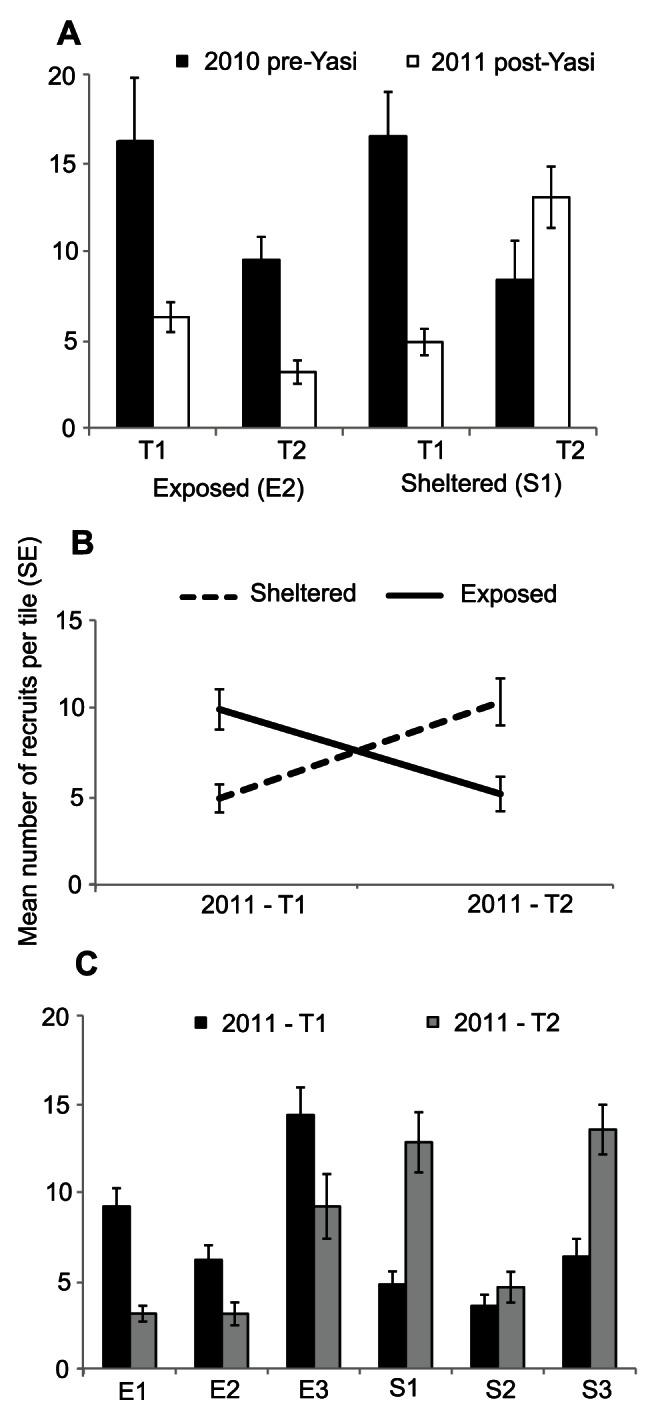
Acroporid recruitment (mean numbers of recruits per tile ± S.E.) at exposed and sheltered sites in the Palm Islands before and after cyclone Yasi. *A.* Acroporid recruitment pre-Yasi (2010) and post-Yasi (2011) at one exposed (E2) and one sheltered (S1) site in each of two temporal windows: the first 14 days post-spawning (T1) and the subsequent month (T2). *B.* Post-Yasi (2011) recruitment in T1 and T2 on exposed and sheltered reefs (mean number of recruits per tile across sites within exposures ± S.E.). *C.* Post-Yasi recruitment at exposed and sheltered sites in two temporal windows. Note the trend of higher recruitment at exposed than sheltered sites in T1 and the reverse trend in T2.

Before cyclone Yasi, the mean number of acroporid recruits tile^−1^ (2010 T1 and T2 combined) did not differ between the exposed (E2: 25.7±4.9 S.E.) and sheltered (S1: 24.9±4.7 S.E.) sites sampled (t-test = 1.407, d.f. = 74, p>0.05). Recruitment after the cyclone (2011 T1 and T2 combined: E2 − 9.4±1.5 S.E. and S1 − 17.9±2.5 S.E.) was significantly lower than recruitment before the cyclone (F_1, 147_ = 9.7, p<0.01, Table 2). The pattern of lower recruitment post-cyclone compared with pre-cyclone also occurred in the first two weeks after spawning (T1) (Fig. 6a). In the second temporal window (T2), recruitment at S1 was also higher after the cyclone than before the cyclone, however the opposite pattern was observed at E2 (Fig. 6a), resulting in a significant interaction between year, time (temporal window) and exposure (F_1, 147_ = 13.8, p<0.001).

**Table 2:** Analysis of variance on density of acroporid recruits at exposed and sheltered sites (Exposure), before and after cyclone Yasi (Year = 2010 & 2011), in two temporal windows (Time = T1 & T2).

Source of Variation	d.f.	MS	F ratio	*P*
Year	1	1.260	9.687	0.002
Time	1	0.130	1.002	0.318
Exposure	1	0.321	2.465	0.119
Year x Time	1	0.688	5.289	0.023
Year x Exposure	1	0.728	5.598	0.019
Time x Exposure	1	0.438	3.368	0.069
Year x Time x Exposure	1	1.796	13.807	0.000
Residual	147	0.130		

*Notes:* Data were log (x+1) transformed.

Following the cyclone (2011), mean recruitment of acroporids in the first two weeks post-spawning (T1) was significantly higher at exposed sites (10.1±1.2 S.E.) than at sheltered sites (5.0±0.8 S.E.) but this trend was reversed in T2 (Fig. 6b), resulting in a highly significant interaction between exposure and time (F_1, 223_ = 66.4, p<0.001, Table 3). There were also significant differences among sites within exposures (F_4, 223_ = 15.7, p<0.001) and this effect was primarily driven by the consistently higher recruitment at E3 than at either of the other two exposed sites, and the lower recruitment at S2 than at the other two sheltered sites (Fig. 6c).

**Table 3:** Nested analysis of variance on post-Yasi density of acroporid recruits at exposed and sheltered sites in two temporal windows (Time = T1 & T2) following spawning in 2011.

Source of Variation	d.f.	MS	F ratio	*P*
Time	1	0.006	0.081	0.777
Exposure	1	0.002	0.021	0.884
Time x Exposure	1	5.039	66.366	0.000
Site(Exposure)	4	1.191	15.687	0.000
Time x Site(Exposure)	4	0.17	2.244	0.065
Residual	223	0.076		

*Notes:* Sites are nested within exposures. Data were log (x+1) transformed.

### Wind Conditions Post-spawning

**Figure 7:**
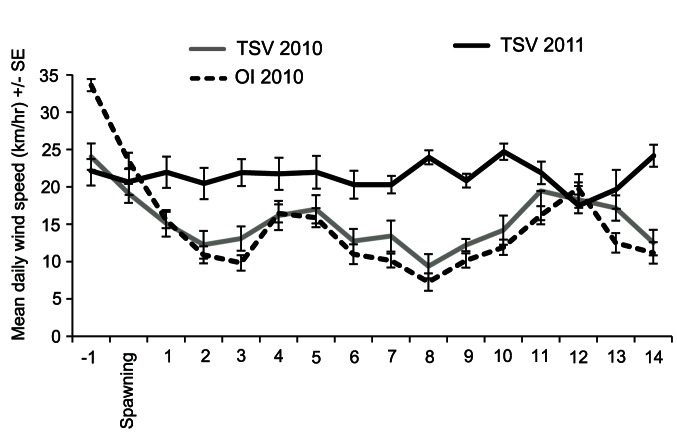
Wind speeds (daily averages of hourly readings ± S.E.) in the first 14 days post-spawning in 2010 and 2011. TSV = Bureau of Meteorology Townsville AERO Weather Station (BOM: 032040): OI = Great Barrier Reef Ocean Observing System Weather Station at Pioneer Bay, Orpheus Island GBROOS: RP3).

In 2010, daily averaged wind speeds recorded at Orpheus Island ranged from 10 to 16 km hr^−1^ in the first week post-spawning and 7 to 20 km hr^−1^ in the second week (Fig. 7). Daily averaged wind speeds at Townsville were very similar to those at Orpheus Island, although typically 2 to 3 km hr^−1^ stronger (Fig. 7), as were the maximum wind speeds (data not shown), suggesting that Townsville wind data provide a good approximation of wind conditions at Orpheus Island. In 2011, daily averaged wind speeds ranged from 18 to 25 km hr^−1^ and were consistently 5 to 15 km hr^−1^ stronger than in 2010 in the first ten days post spawning (Fig. 7). In 2011, maximum daily wind gusts [average (± S.E.) of the five strongest wind gusts recorded per 24 hours] ranged from 37.8 (±0.9) to 41.2 (±1.4) km hr^−1^, whereas in 2010, maximum wind gusts were typically 10 to 12 km hr^−1^ weaker, ranging from 26.6 (±1.7) to 33.2 (±1.4) km hr^−1^.

## Discussion

Coral reefs around the world are being increasingly impacted by multiple stressors, which have reduced coral cover and degraded reef ecosystems [44], yet there is currently no consensus about the long-term trends in coral cover for the GBR. Some studies have demonstrated ecosystem-wide declines [31], [45], [46] while others have reported reasonably stable coral cover across the entire GBR, in the face of asynchronous fluctuations among subregions [32], [47]. Nonetheless, two recent studies that quantified the relative contributions of major disturbances (cyclones, coral bleaching, and predation by crown-of-thorns starfish, *Acanthaster planci*) on the GBR demonstrated that tropical cyclones account for large proportions of coral loss [31], [32]. Specifically, [31] found that tropical cyclones accounted for 48% of coral mortality on the GBR between 1985 and 2012, during which time total coral cover declined by 50% (from 28% to 14%), while [32] reported that cyclones caused 34% of regional losses in coral cover between 1995 and 2009, mostly due to their impacts on *Acropora* (although they found no net coral loss on the GBR during this time).

Cyclones rarely reduce coral cover to zero. Damage is typically spatially heterogeneous, depending on the characteristics of the cyclone (e.g. size, wind speed, duration, wave-heights) and the reef habitats involved (e.g. location, reef size and orientation, coral community) [29], [34]–[36]. Cyclone Yasi severely impacted coral populations on the exposed (eastern) sides of Orpheus and Pelorus Islands but did not similarly impact sheltered sites. Rapid ecological assessments conducted in the months after cyclone Yasi found similarly high spatial variation in damage on 76 reefs in the cyclone’s path [28]. In addition to being spatially heterogenous, cyclone damage is typically selective, targeting vulnerable species groups or colony morphologies [48], in particular the Acroporidae [32]. Cyclone Yasi reduced *Acropora* cover to <0.1% of all benthic habitats at exposed sites of the Palm Islands, and the surviving colonies of *Acropora* were very small (most <5 cm, all <10 cm) and consisted mostly of encrusting bases with a few small branches. *A. tenuis* juveniles reach 1 cm in diameter at six months of age, and 3 to 5 cm by 1.5 to 2.5 years [49], but acroporids typically need to reach sizes of 10–15 cm diameter before they are reproductively mature [15], [50], [51]. Thus, the size and colony morphology of *Acropora* at exposed sites nine months after cyclone Yasi indicated that only immature colonies survived the cyclone. It will be several years before these juveniles reach reproductive maturity and contribute to the larval pool.

The genus *Acropora* is the most abundant and species-rich scleractinian genus on the GBR and on reefs throughout the Indo-Pacific [16], [52] so recovery of *Acropora* assemblages is essential for the long-term persistence of coral reefs in this region. In the GBR (and the Indo-Pacific), recovery of local *Acropora* assemblages following disturbance relies heavily on replenishment by planktonic larvae [15]. This is unlike the Caribbean, where recovery of the two major reef-building *Acropora* species relies predominantly on regrowth of live fragments [6]. Our study found that, despite the absence of reproductively mature colonies of *Acropora* at exposed sites following cyclone Yasi, recruitment to settlement tiles did not drop to zero, indicating that impacted sites were receiving larval subsidies from external sources in the first spawning event after the cyclone. These findings are consistent with studies demonstrating that the relationship between acroporid abundance and larval supply operates at spatial scales larger than a single reef in the GBR [26], [53]. Similarly, population genetics indicated high connectivity (panmixia) for *A. millepora* across inshore and mid-shelf reefs in the northern and far northern GBR [24], consistent with larval dispersal among reefs, although some reefs (mostly in the southern GBR) were predominantly self-seeding [24].

Coral fecundity can be reduced by stress [54], and large-scale temporal (and spatial) variation in acroporid recruitment to settlement tiles on the GBR has been found to be more strongly associated with fecundity than adult abundance [26]. Indeed, much lower acroporid recruitment to experimental substrata (<2 recruits tile^−1^) was recorded at the Palm Islands nine months after the 1998 mass-bleaching event [55], which caused widespread mortality of acroporids in the central GBR (e.g. 90–95% mortality of *Acropora* colonies at Orpheus Island) [56], [57] and reduced coral fecundity [55]. Recruitment to settlement tiles following cyclone Yasi did not drop to these low levels, potentially suggesting that sub-lethal stressors did not significantly reduce fecundity following the cyclone. Although recruitment to settlement tiles did not drop to zero at impacted sites, acroporid recruitment in the Palm Islands following the cyclone was significantly lower overall than in the year before the cyclone (Fig. 6a). Cyclone Yasi damaged or destroyed coral populations on many central GBR reefs [28], which is expected to have reduced the 2011 larval pool in this region. Thus, it is likely that the reduced larval supply observed at the Palm Islands also occurred at other reefs, particularly those closer to the eye of the cyclone that suffered more severe damage than the Palm Islands [28].

*Acropora* larvae typically have a pre-competency period of two to three days, after which they begin to search for suitable substratum, and settle between four to seven days post-spawning [17]–[19]. During this time, larvae are in the water column and subject to complex local hydrodynamic features (such as tides and eddies) that influence local retention and self-seeding, and also to wind-driven surface currents that promote dispersal away from the reef [23], [58]. Hydrodynamic models indicate that retention times of coral larvae on reefs can vary from hours to days, depending on local wind conditions [59]. Similarly, empirical studies found that during quiescent conditions (wind speed 8–20 km hr^−1^), coral larvae formed slicks that were visible for up to 22 hours as they dispersed away from natal reefs, whereas under strong wind conditions (>25 km hr^−1^) coral larvae were quickly dispersed throughout the water column [43]. Following the cyclone (2011), recruitment to settlement tiles in the first two weeks post-spawning (T1) was higher at impacted, exposed sites than at sheltered sites (Fig. 6b, c), indicating that the initial larval supply came from distant sources. This result is consistent with the strong winds recorded immediately following spawning in 2011 (Fig. 7), which can be expected to have transported larvae away from the sheltered Palm Island reefs via surface currents. Once coral larvae are moved away from the reef, dispersal is driven primarily by meso-scale processes, which appear to have brought more larvae to sheltered than exposed sites during subsequent weeks (2011_T2) (Fig. 6b, c). By contrast, in 2010 the initial recruitment pulse (T1) was higher than in subsequent weeks (T2) at both exposed and sheltered sites (Fig. 6a), consistent with the calmer conditions in 2010 in the first week after spawning (Fig. 7).

### Caveats and Limitations of the Study

Coral recruitment is notoriously variable in space and time [26], [29], [41], [60] and large annual variation in recruitment of *Acropora* to settlement tiles has been documented for the GBR in the absence of an obvious disturbance [51]. Unfortunately, recruitment data were only available for one year prior to the cyclone at one sheltered and one exposed site, thus do not provide information on natural spatial and temporal variability in recruitment before the disturbance. Nonetheless, the fact that recruitment to settlement tiles did not drop precipitously at impacted sites in the first spawning event following the cyclone provides important information about the potential for larval supply to reseed these decimated reefs. There were also no data for benthic cover before the cyclone, however, extensive fieldwork conducted at exposed and sheltered reefs in the Palm Islands in 2010 (by VL, GT and BW) indicated that coral cover, diversity and structural complexity at exposed reefs was as high, if not higher, than at sheltered reefs immediately before the cyclone (see Fig. 5). These observations are supported by the abundance and size data collected for *A. tenuis* at one exposed site in 2010 (Fig. 4b).

This study quantified larval recruitment to experimental substrata, which is the most commonly used approach for estimating coral recruitment [7], [26], [41], [51], [53], [60]–[62]. While this approach provides a reasonable indication of larval supply, it does not estimate successful recruitment to natural habitats. Previous studies in the Caribbean have documented lower recruitment to natural habitats by non-branching corals in years of severe tropical storms by modeling growth rates of corals [37]–[39]; however, this approach is not possible with branching *Acropora* corals because of their potential to reproduce by fragmentation [6], [7]. A study to monitor the appearance of recruits in natural habitats on impacted reefs over the coming years (using fixed transects) is being undertaken.

Inferences about larval dispersal based on wind data from 2010 and 2011 provided support for the observed patterns of recruitment, however, a more sophisticated approach would be to evaluate the potential distance and direction of larval dispersal using particle tracking models and hydrodynamic data [63]. The validity of such an approach relies on hydrodynamic data (from the relevant time periods) with sufficiently high spatial resolution to resolve the fine-scale hydrodynamic processes that occur in the complex shallow-water bathymetry of the GBR [64]. Such hydrodynamic models are currently being developed for the GBR [64] and should prove useful for evaluating larval dispersal patterns in the future.

### Long-term Trends and the Potential for Recovery of Reefs of the GBR Following Cyclone Yasi

Time-series data documenting the recovery of reefs following outbreaks of *A. planci* on the GBR between 1989 and 1994 demonstrated that heavily damaged areas had the capacity to recover through larval settlement, recruitment and growth, and that *Acropora* contributed proportionally more to hard coral cover as recovery progressed [65]. Importantly, juvenile colonies were a significant component of the *Acropora* assemblage five to eight years after the disturbance, indicating that larval supply had contributed substantially to recovery [65]. Similarly high recovery rates occurred between 1995 and 2009 following disturbances at reefs dominated by *Acropora* [32] suggesting that recruitment and growth of *Acropora* had kept up with the effects of disturbances on most reefs (although corals with less capacity for recruitment and growth than Acroporidae had widespread negative trends) [32]. Nonetheless, recovery rates after cyclones were lower than after *A. planci* infestations [32], possibly due to the loss of structural complexity following cyclones [66]. Moreover, the positive trends in recovery seen following the *A. planci* infestations in the early 1990s were not seen in in the early 2000s on reefs impacted by both *A. planci* and coral bleaching in 1998 (or 2002) [65]. Of particular concern was the small numbers of juvenile *Acropora*, suggesting that there had not been a strong recruitment pulse post-disturbance [65], likely to be the result of reduced fecundity caused by sub-lethal bleaching [54].

Recent paleoecological evidence suggests that *Acropora* assemblages in the Palm Islands suffered a major collapse between 1920 and 1955 [67]. Nonetheless, in 1996, live hard coral cover on exposed Palm Island reefs was consistently higher than 40% and often more than 60% [55] and evidence from historical photographs suggests that in the years immediately preceding the 1998 mass-bleaching event, coral assemblages on reef flats in the Palm Islands looked very similar to those photographed 100 years earlier [68]. The 1998 mass-bleaching event severely reduced coral cover and diversity on Palm Island reefs [56], [57] and recovery was probably interrupted by a subsequent bleaching event in 2002 and cyclone Larry in 2006. Genetic studies have shown that recovery following the 1998 bleaching event brought an influx of new genetic material, which changed the genetic characteristics of some *Acropora* populations in the Palm Islands [24], [69], highlighting the importance of larval subsidies in the recovery process, although there was also genetic evidence of re-growth of surviving colonies [69]. Cyclone Yasi caused major structural damage to reefs [28] and removed all adult colonies and fragments of *Acropora* from exposed Palm Island reefs. It is unlikely that regrowth of remnant live coral tissues will contribute substantially to recovery of coral communities impacted by cyclone Yasi, particularly as structural complexity has been shown to be important for rapid reef recovery [4]. Our findings that larval recruitment to experimental substrata occurred at impacted sites in the first spawning event following the disturbance, combined with the presence of juvenile colonies of *Acropora* at those sites, suggest that these impacted populations of *Acropora* have the potential to recover over time. However, if this relatively slow recovery process is again interrupted by disturbance (such as cyclones, bleaching or *A. planci* outbreaks) or hindered by other stressors (such as poor water quality, pollution, disease, or reduced herbivore numbers resulting in macroalgal overgrowth of young corals), coral populations in the Palm Islands may fail to recover, with the consequence that reefs will undergo phase-shifts to less desirable algal-dominated states [10], [11], [70].
